# Consensus statement from the first international conference for *in utero* stem cell transplantation and gene therapy

**DOI:** 10.3389/fphar.2015.00015

**Published:** 2015-02-10

**Authors:** Tippi C. MacKenzie, Anna L. David, Alan W. Flake, Graca Almeida-Porada

**Affiliations:** ^1^Eli and Edythe Broad Institute for Regeneration Medicine, University of California, San FranciscoSan Francisco, CA, USA; ^2^Institute for Women's Health, University College LondonLondon, UK; ^3^Children's Hospital of PhiladelphiaPhiladelphia, PA, USA; ^4^Wake Forest Institute for Regenerative Medicine, Wake Forest UniversityWinston-Salem, NC, USA

**Keywords:** *in utero* transplantation, *in utero* gene therapy, fetal tolerance, Hematopoietic Stem Cells, Stem Cell Niche

On April 17–18, 2014, basic and translational scientists and clinicians convened in San Francisco, CA for a conference in fetal stem cell transplantation, stem cell biology, tolerance, and gene therapy.

The purpose of the meeting (http://pedsurglab.surgery.ucsf.edu/news–events/fetal-symposium-2014.aspx) was to outline the goals of *in utero* transplantation, review the barriers that have been encountered, and learn about new developments that can be applied to the field.

Information discussed at this conference will help pave the way for developing novel strategies to achieve therapeutic engraftment levels in the fetus, and identify ways to safely translate these strategies to a wide range of clinical applications.

We held a final consensus session to achieve an international agreement for future pre-clinical and clinical studies of *in utero* hematopoietic cell transplantation (IUHCT). We agreed on the following items:

- *In utero* transplantation is a viable strategy to treat fetuses with selective congenital disorders.
- Given recent publications that the maternal immune response can limit engraftment, the clinical strategy for IUHCT should involve transplantation of autologous or maternal-derived cells. The host immune response may be a limiting factor that might be circumvented with early cell delivery.
- The fetal microenvironment plays a primary role in supporting the engraftment and expansion of transplanted cells and requires further investigation.
- Recent data from large animal studies suggests that intravascular injection may be the delivery route of choice to achieve engraftment of hematopoietic stem cells in the fetus.
- Currently, there is no proven safe method of host conditioning in the fetus. Until specific, non-toxic conditioning methods (such as antibody-mediated depletion of host HSC) are optimized in pre-clinical models, large cell doses should be used to overcome host competitive barriers.
- Experimental model data are sufficient to warrant a phase 1 clinical trial of IUHCT for select fetuses. The most suitable hematological diseases are hemoglobinopathies such as sickle cell disease and thalassemia, given their high morbidity/mortality, the availability of reliable prenatal screening programs, and the paucity of optimum postnatal care options.
- The value of alternative cells, such as mesenchymal stromal cells (MSC) and amniotic fluid-derived cells, for other appropriate congenital pathologies warrants investigation.
- Reports of using MSC *in utero* to treat osteogenesis imperfecta (OI) in a limited number of patients are promising and suggest that, after optimization, MSC could be used to improve/treat OI.
- Treatment of the fetal patient using gene therapy and gene-modified cells have great future potential and should be fields of active investigation.
- A new society focused on fetal stem cell transplantation and gene therapy will be formed (FeTIS: Fetal Transplantation and Immunology Society), with the mission of accelerating clinical applications of stem cell transplantation and gene therapy approaches to treat fetuses with congenital anomalies.
- The society should develop and maintain an international registry of treated patients and their outcomes to facilitate reporting and sharing of results. This database will not be publicly accessible and data will be anonymized.
- The society will provide a forum for members to share best practice and clinical governance for *in utero* stem cell and gene therapy cases.

## Conflict of interest statement

The authors declare that the research was conducted in the absence of any commercial or financial relationships that could be construed as a potential conflict of interest.

